# Whole-cell hydrolysate of cyanobacteria enhances biofuel fermentation of plant biomass

**DOI:** 10.1093/sumbio/qvaf007

**Published:** 2025-05-01

**Authors:** Valentina L Donati, Benedikt M Blossom, David Cannella, Niels-Ulrik Frigaard

**Affiliations:** Department of Biology, University of Copenhagen, 3000 Helsingør, Denmark; Department of Biology, University of Copenhagen, 3000 Helsingør, Denmark; Department of Geosciences and Natural Resource Management, University of Copenhagen, 1958 Frederiksberg C, Denmark; Department of Geosciences and Natural Resource Management, University of Copenhagen, 1958 Frederiksberg C, Denmark; Department of Biology, University of Copenhagen, 3000 Helsingør, Denmark

**Keywords:** biofuels, wastewater, revalorization of lignocellulosic biomass, fermentation biotechnology, cyanobacteria

## Abstract

Biofuel fermentation of plant materials by yeast typically requires supplementation with nutrients, such as vitamins and nitrogenous compounds, to enable yeast metabolic activity and growth. In this study, we used a simple milliliter-scale fermentation vial system to demonstrate that hydrolyzed whole-cell biomass from a photosynthetic cyanobacterium (*Synechococcus* sp. PCC 7002) can be used as a nutrient supplement for yeast fermentation of wheat straw and spruce. Adding lysozyme-treated, high-nitrogen cyanobacterial biomass (0.5% dry matter, w/v) to a *Saccharomyces cerevisiae* fermentation of wheat straw hydrolysate (∼30% dry matter, w/w) increased the initial ethanol production rate from 6.7 ± 0.2 to 18.7 ± 1.0 g l^−1^ day^−1^, representing a nearly three-fold enhancement. Supplementation of an equivalent amount of acid-hydrolyzed cyanobacterial biomass increased ethanol production rates by only ~50%, indicating that essential nutrients were degraded during acid treatment. In addition, high-nitrogen cyanobacterial biomass improved initial ethanol production rates from wheat straw by 30%–80% compared to low-nitrogen biomass, suggesting that nitrogenous nutrients in the cyanobacteria were responsible for the enhanced yeast activity. Our data show that biomass from cyanobacteria can be used as a sustainable alternative to conventional nutrient supplements for biofuel fermentation of lignocellulosic biomass, including wheat straw and spruce hydrolysates.

**Sustainability Statement**

This study demonstrates that photosynthetically produced cyanobacterial biomass can replace conventional nutrients in biofuel fermentation of plant-derived biomass, thereby improving efficiency and promoting resource reuse. This supports the UN Sustainable Development Goals (SDG) of Affordable and Clean Energy (SDG 7) by enhancing biofuel production, Responsible Consumption, and Production (SDG 12) by utilizing biological waste sustainably, Climate Action (SDG 13) by reducing reliance on fossil fuels, and Life on Land (SDG 15) by decreasing the need for land-intensive biofuel crops.

## Introduction

In order to minimize greenhouse gas emissions and to fuel transportation systems in a carbon-neutral manner, sustainable solutions are required to replace fossil fuels (Köne and Büke 2010, Cherwoo et al. 2023, El-Araby 2024). In this context, the concept of biorefineries is a promising approach, processing renewable resources into advanced fuels, such as bioethanol, and a wide range of other marketable products (Annevelink et al. 2022, Calvo-Flores and Martin-Matinez 2022, Pérez-Almada et al. 2023, Long et al. 2025). Unlike crude oil refineries, biorefineries rely on the stepwise refinement of different types of biomass into their molecular components. Through photosynthesis, plants, algae, and cyanobacteria collect and transform solar energy into chemical energy, which is stored in a wide range of biomolecules, including sugars, cellulose, lignin, and oils. The complete refinement of biomass from these organisms—for example, the conversion of microbial biomass or plant material into their basal components—includes a broad range of technologies aiming at producing biofuels (e.g. bioethanol, biodiesel, and biogas) and value-added products such as nutritional supplements (e.g. pigments, antioxidants, and polyunsaturated fatty acids) and platform chemicals (e.g. lactic acid, succinic acid, and furfural) (Calvo-Flores and Martin-Matinez 2022, Pérez-Almada et al. 2023).

Advanced biofuels are based on a wide range of non-food crops, implementing a variety of lignocellulosic materials, such as residues from forestry, industry, agriculture, and dedicated lignocellulosic crops as feedstocks (Rubin 2008, Faraco 2013, Broda et al. 2022). The main components of lignocellulosic materials are *cellulose*, a homo-polysaccharide made of d-glucose molecules and the main building block of the plant cell wall; *hemicellulose*, a heterogeneous group of complex polysaccharides; and *lignin*, the main non-polysaccharide fraction (Taiz and Zeiger 2006; Jørgensen et al. 2007). To produce biofuels from lignocellulosic material, a pre-treatment step is crucial to disrupt the complex plant cell wall structure before the enzymatic hydrolysis of cellulose (Bacovsky et al. 2010, Galbe and Wallberg 2019). However, these steps also release compounds that negatively affect subsequent microbial fermentations. For instance, furfurals and 5-hydroxymethyl furfurals (HMFs) are produced from the degradation of xylose and hexoses, respectively, while the partial depolymerization of lignin produces phenolic compounds (Palmqvist and Hahn-Hagerdahl 2000). In order to detoxify the treated lignocellulosic materials, several enzymatic, physical, and chemical strategies can be employed (Chandel et al. 2011). Furthermore, genetic modifications of the yeasts have been studied in depth to enhance their resistance to fermentation inhibitors (Larsson et al. 2001, Koppram et al. 2012). It is also possible that certain synthetic or biological supplements could mitigate the adverse effects of fermentation inhibitors, either by inactivating the inhibitors or by conferring the yeast with increased resistance (Ujor and Okonkwo 2022).

A nutrient source such as yeast extract, peptone, or ammonium salts is usually supplemented in yeast fermentations of carbohydrates or plant-derived lignocellulosic biomass to compensate for the absence, or low content, of nitrogen and other nutrients in these substrates (Jørgensen 2009). The full range of micronutrients required for optimal yeast growth and fermentation is not clear, but it likely is a complex mixture of various vitamins, minerals, and other growth factors (Hahn-Hägerdal et al. 2005). Perli et al. (2020) attempted to map the requirements for vitamins in *Saccharomyces cerevisiae* based on genome sequence analyses. Experimental studies by Alfenore et al. (2002) and Wang et al. (2024) found that specific vitamins enhance the ethanol yield during glucose fermentation by *S. cerevisiae* in a mineral medium. Van Dijk et al. (2020) also found that vitamin supplements in *S. cerevisiae* fermentations of wheat straw hydrolysate significantly increase ethanol production, again demonstrating the importance of nutrient supplementation for improving fermentation efficiency. Jørgensen (2009) tested *S. cerevisiae* fermentation of wheat straw hydrolysate with different nitrogen sources, including urea, peptone, (NH_4_)_2_SO_4_, corn steep liquor, and yeast extract. The results showed that yeast extract (at a content of 3.5 g kg^−1^) was the best supplement to enhance fermentation productivity (0.6 g kg^−1^ h^−1^). However, despite its beneficial effects, yeast extract is an expensive supplement, and cheaper alternatives are desirable for large-scale fermentations. Yeast extract and peptones are the most commonly reported microbial-derived nutrient supplements used in biofuel fermentations, and to our knowledge, there are no reports on the use of other microbial biomass hydrolysates solely as a source of nutrients for ethanol fermentation by yeast. Several studies have shown that hydrolysates of carbohydrate-rich biomass from microalgae and cyanobacteria can be fermented to ethanol by yeast (e.g. Ho et al. 2013, Markou et al. 2013, Möllers et al. 2014, Huang et al. 2018, Silva et al. 2018, Tsolcha et al. 2021), but it is not clear from these studies to what extent these hydrolysates contribute essential nutrients beyond fermentable carbohydrates.

Aquatic phototrophs, most notably the prokaryotic cyanobacteria and the eukaryotic microalgae, are promising feedstocks for the production of advanced biofuels (Schenk et al. 2008, Dragone et al. 2010, Slade and Bauen 2013). These organisms convert CO_2_ and inorganic nitrogen into organic matter using natural or artificial light as the source of energy. In some cases, growth can be enhanced by supplementation with simple organic compounds such as glycerol or acetate, which can be obtained as byproducts from biodiesel production and other industrial waste streams (Zhan et al. 2017). Depending on the cultivation conditions, more than 50% of the biomass of these organisms may consist of carbohydrates or lipids, which can be converted into biofuels by chemical treatments and/or microbial fermentations. These photosynthetic microbes offer several advantages over plants and macroalgae as substrates for biofuel production, including faster growth, higher efficiency of solar energy conversion, absence of recalcitrant biopolymers, metabolic and ecological diversity, and the possibility to biosynthesize desired compounds using environmental and nutritional stresses (Dismukes et al. 2008). Photosynthetic microbes also have the advantage that they can be cultivated in many types of wastewater, thereby removing nutrients from the wastewater that might otherwise pollute the environment and simultaneously converting the wastewater nutrients into useful bioproducts (Calatrava et al. 2024). The biomass of cyanobacteria and certain green microalgae is often rich in carbohydrates, especially if the cells grow under nitrogen-limiting conditions, and therefore this biomass is suitable as a carbon feedstock for yeast fermentation for the production of bioethanol (e.g. Ho et al. 2013, Markou et al. 2013, Möllers et al. 2014). In contrast, if the cells are cultivated under nitrogen-replete conditions, the cells typically accumulate high amounts of nitrogenous compounds and less of carbon storage compounds. The carbon to nitrogen (C/N) ratio in the biomass, which is dependent on the relative contents of polysaccharides, nucleic acids, lipids, and proteins (Raven et al. 2004), is ∼4–5 in cyanobacteria cultivated under optimal and nitrogen-replete conditions, as compared to 12–14 in cyanobacteria cultivated under nitrogen-limiting conditions (Möllers et al. 2014, Huang et al. 2018), 6–11 in microalgae (Finkel et al. 2016), 10–40 in macroalgal seaweed (Duarte 1992), and 50–100 in wheat straw (Reichel et al. 2018). These variations in the C/N ratios imply a potential for a higher nitrogen assimilation in cyanobacteria than in most other phototrophic organisms, which could make cyanobacteria a rich source of sustainable and natural nitrogenous compounds that may replace expensive supplements such as yeast extract in microbial fermentations.

In this study, we investigated the use of cyanobacterial biomass as a nutrient supplement for ethanol fermentation of glucose and lignocellulosic biomass. The importance of the cyanobacterial biomass was primarily as a source of nitrogenous compounds and micronutrients, and not as a significant source of fermentable carbohydrates. The main source of fermentable carbohydrates in our experiments was either pure glucose or carbohydrates derived from treated plant biomass.

## Materials and methods

### Cultivation and treatment of *Synechococcus* sp. PCC 7002

*Synechococcus* sp. PCC 7002 (received from Donald A. Bryant, The Pennsylvania State University, PA, USA) was cultivated in medium A+ (Stevens et al. 1973) with CO_2_ and nitrate as the sole sources of carbon and nitrogen, respectively, as previously described (Möllers et al. 2014). Medium A+ has a pH of ca. 8.0 and contains 1 g l^−1^ of Tris buffer (CAS 77–86-1). Cultivation for biomass preparation for fermentation assays was performed in 1.8-l culture flasks (inner diameter 10 cm) placed in a 30-l transparent water bath thermostatic controlled at 38°C and under continuous illumination by cool white fluorescent light (Philips Master TL-D, 18 W/840; Philips Electronics; Amsterdam; The Netherlands) with an intensity of 250 μmol photons m^−2^ s^−1^ at the surface of the culture flask. The cultures were continuously stirred magnetically (ca. 400 rpm) and aerated (ca. 1 vol of gas per 1 vol of liquid per min) with air containing 1% v/v CO_2_ provided by a gas mixer (GMS150; Photon Systems Instruments, Drasov, Czech Republic). Nitrogen-replete condition was obtained using 2.0 g NaNO_3_ l^−1^ and nitrogen-limited condition using 0.24 g NaNO_3_ l^−1^. pH was not controlled during growth and was ∼8.5–9 at the time of harvest. Biomass was harvested after around 65 h cultures at an optical density at 730 nm (OD_730_) of ∼4 by centrifugation (8000 rpm for 45 min at 4°C using a JA-10 rotor and an Avanti J-26 XP centrifuge; Beckman Coulter, Brea, CA, USA). After having discarded the supernatant, cell pellets were resuspended in water (purified by Milli-Q system), transferred to 50-ml Falcon tubes, the water removed by centrifugation, and the remaining cell pellets stored at −20°C until use. The thawed cyanobacterial biomass was briefly sonicated (Misonix S-4000; Qsonica, Newtown, CT, USA) with a cup horn sonicator (amplitude 100%, processing time 2 min, on/off cycle 5 seconds) to loosen up aggregated cell material and then treated in different manners to release cellular compounds (see below). A UV1800 spectrophotometer (Shimadzu, Kyoto, Japan) was used for optical measurements and determination of the chlorophyll and phycobilisome (PBS) content in *Synechococcus* cultures as previously (Möllers et al. 2014).

Acid hydrolysis is a common pretreatment to mobilize fermentable sugars from microbial and plant biomass and is widely used to generate monosaccharides from algal sugar polymers, such as starch and glycogen, to be subsequently converted by yeasts (Markou et al. 2013). Here, the treatment was used to liberate nutritious compounds, and the released glucose content was used as a measure of cell disintegration. The cyanobacterial biomass was treated with 2% w/v H_2_SO_4_ for 3 h at 100°C after which the pH was adjusted to pH 5 with 2 M NaOH.

Lysozyme causes lysis of cyanobacterial cells because it disintegrates their cell wall by hydrolysis of the peptidoglycan layer (Möllers et al. 2014). Lysozyme (200 mg l^−1^; from chicken egg white, L6876, Sigma-Aldrich, St. Louis, USA) was added to the cyanobacterial biomass (pH 8–9), and the suspensions were incubated for 24 h at 37°C. Alternatively, after incubation with lysozyme for 4 h at 37°C, the temperature was increased to 50°C for 20 h. Biomass treatments with acid and lysozyme were not combined.

### *Saccharomyces cerevisiae* cultivation and fermentation setup

*Saccharomyces cerevisiae* strain Thermosacc® dry (Lellemann, Canada) was used for all fermentation experiments and pre-cultivated in 50 l CBS (Centraalbureau voor Schimmelcultures) medium (Verduyn et al. 1992). Filter-sterilized vitamins were added to the autoclaved CBS medium to final concentrations of biotin, 0.05 mg l^−1^; calcium pantothenate, 1 mg l^−1^; nicotinic acid, 1 mg l^−1^; inositol, 25 mg l^−1^; thiamine HCl, 1 mg l^−1^; pyridoxine HCl, 1 mg l^−1^; and *para*-aminobenzoic acid, 0.2 mg l^−1^. The medium was also supplemented with glucose, 20 g l^−1^, as a carbon source and then incubated for 48 h at 35°C (100 rpm). After the incubation, cells were harvested through centrifugation (5 min at 3000 g), re-suspended in 5 ml of the supernatant (concentrated solution of 75 g kg^−1^) and used as inoculum for the fermentation experiments to have a final concentration of 1.2 g kg^−1^ (or 6 g kg^−1^ WIS content). Yeast fermentations were performed in 10 ml glass vials with a broth volume of 3 ml. The vial was sealed with a pressure-resistant rubber stopper and a needle placed through the stopper. The different fermentation substrates were inoculated with concentrated yeast suspension. The fermentation vials were then incubated in an orbital shaker (100 rpm) at 35°C for the time needed to have a complete fermentation. Measurements of the weight of the vials were performed throughout the experiment. The weight loss of the vials was used as a measure of the production of CO_2_, and from this, ethanol production and glucose consumption during the fermentation were derived using the following equations:


\begin{eqnarray*}
\begin{array}{@{}l@{}}
\text{Consumed glucose}\,\,(\mathrm{g}\ \mathrm{l}^{-1})
= \left( \frac{\mathrm{CO}_2\ \text{loss (mg)}\, \cdot\, 2.05} {3\ \mathrm{ml}} \right),\\
\text{Produced ethanol}\,\,(\mathrm{g}\ \mathrm{l}^{-1})
= \left( \frac{\mathrm{CO}_2\ \text{loss (mg)}\, \cdot\, 1.05} {3\ \mathrm{ml}} \right).
\end{array}
\end{eqnarray*}

To assess the effect of the cyanobacterial biomass as a nutrient supply during the fermentation of wheat straw and spruce, 10 ml stocks were prepared with the desired amounts of the selected components, and the pH was then adjusted to 5 by using H_2_SO_4_ (72% w/v). The mixtures were subdivided into three fermentation vials. The yeast was then inoculated in each vial, which was then closed and incubated as described above. The vial head space was not flushed with any gas. Fermentation activity in CBS medium was always used as a control of yeast viability. Due to the possible influence of water evaporation on the vial weight loss, a negative control was also always performed where the yeast inoculum was substituted with water. Fermentations were always performed in biological duplicates or triplicates.

### Wheat straw material for fermentation

Wheat straw (*Triticum aestivum* L.) was hydrothermally treated at Inbicon IBUS pilot plant (Skærbækværket, Skærbæk, Denmark). Soaking in water (80°C, 5–10 min) was the first step of the pre-treatment performed prior to transportation in the reactor where the material was hydrothermally treated (195°C by steam injection for 12 min) without the addition of chemicals. The final dry weight of the material, which was collected in plastic bags and stored at 4°C, was around 40% w/w. Cellic CTec2, a commercial cellulase preparation (Novozymes A/S, Bagsværd, Denmark) with an activity of 120 FPU g^−1^ was added to the pre-treated wheat straw at a dosage of 22.8 mg per g of dry matter together with Na-citrate buffer (50 mM, pH 4.8) and water. The mixture, contained in a 100 ml blue cap bottle, was hydrolyzed for 4 days in an orbital shaker (150 rpm) at 50°C (Jørgensen 2009). The obtained hydrolysate was harvested through centrifugation, and the liquid and the solid fractions were separated and stored at 4°C.

### Spruce material for fermentation

The spruce wood material (chips) was pretreated at the SEKAB pilot plant (Ӧrnskӧldsvik, Sweden) (temperatures at around 210°C–215°C, 5–7 min as average residence time, SO_2_ as catalyst). After the process, the dry weight of the spruce was around 30%, and the pH was around 2. The slurry (pH 1) placed in 100 ml blue cap bottles was adjusted to pH 5 with the addition of 13 M NaOH (5 ml). Cellic CTec2 was added to the slurry at a dosage of 22.8 mg per g of dry matter. The mixture was then hydrolyzed for 5 days in an orbital shaker (150 rpm) at 50°C (Cannella et al. 2014). The obtained hydrolysate was harvested through centrifugation and the liquid and the solid fractions were separated and stored at 4°C. In order to remove furans and aliphatic acids (yeast inhibitors), polyethylenimine (PEI), a soluble polyelectrolyte polymer, has been utilized for the detoxification of spruce from recalcitrant biopolymers. Indeed, this cationic polymer can selectively adsorb yeast inhibitors without co-removal of sugars. The detoxification of the hydrolysate was performed with the addition of linear PEI solutions (50% w/w in H_2_O, 60,000 average MW, Sigma Aldrich) directly to the harvested liquid fraction. H_2_SO_4_ (72% w/v) was subsequently used to adjust the pH to allow the fermentation (pH 5) (Cannella et al. 2014).

### HPLC analysis of soluble low-molecular-weight compounds

High-performance liquid chromatography (HPLC) analysis was performed to quantify monosaccharides (d-glucose, l-arabinose, d-xylose), ethanol, lactate, acetate, HMF, and furfural. An Ultimate 3000 HPLC (Dionex, Germering, Germany) equipped with a UV detector at 210 and 280 nm (Dionex) and an RI-detector (Shodex, Japan) was used. A Rezex ROA column (Phenomenex, Torrance, CA, USA) at 80°C with 5 mM H_2_SO_4_ as eluent at a flow rate of 0.6 ml min^−1^ was used to perform separation. Samples were diluted 10-fold with 0.1 M HCl and mixed. Insoluble materials were removed by centrifugation at 4.000×*g* for 3 min, and the supernatant was then filtered (0.45 µm) and analyzed by HPLC.

### Statistics

GraphPad Prism version 10.4.1 for Windows (GraphPad Software, La Jolla, CA, USA, www.graphpad.com) was used for linear regression and statistical analyses. For ANOVA, normality was tested using the Shapiro–Wilk test, and homogeneity of variances was assessed using Levene’s test (for datasets with more than two replicates; in the case of duplicates, homogeneity was assumed). Comparisons of ethanol production rates between different fermentation setups were performed using one-way ANOVA, followed by Dunnett’s post hoc test for multiple comparisons against the control fermentation. *P*-values below .05 were considered statistically significant.

## Results

### Setup and reliability of the fermentation system design

**Figure 1.**
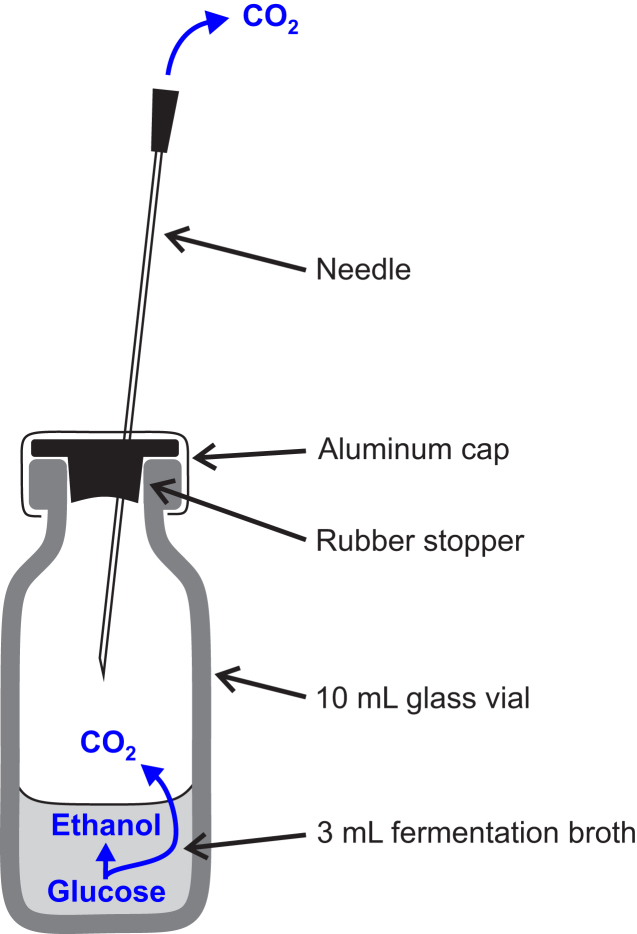
Diagram of the small-scale fermentation vial setup for ethanol production assays. During the fermentation process, glucose is metabolized by *S. cerevisiae* into ethanol and CO_2_. CO_2_ passes through the needle, causing a measurable weight loss of the vial, which correlates with the amount of ethanol produced. The needle also restricts entry of O_2_, thus maintaining anaerobic conditions.

**Figure 2.**
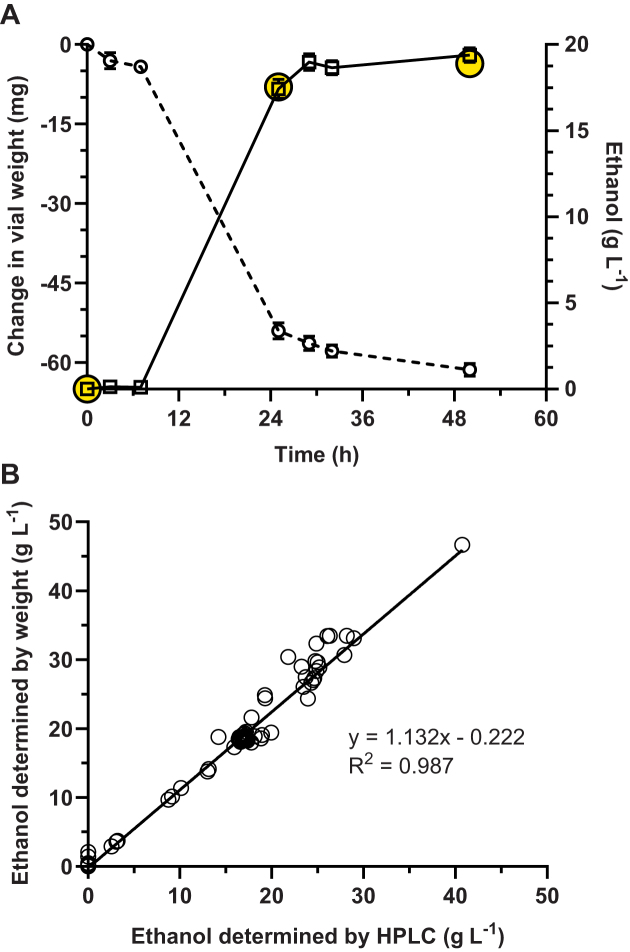
Validation of the fermentation system design. (A) Vial weight loss (open circles and dashed lines) and calculated ethanol production (open squares and full lines) in CBS medium with glucose (40 g l^−1^) by *S. cerevisiae*. The ethanol produced was estimated from the vial weight loss (see the “Materials and methods” section). The ethanol concentration determined by HPLC in the same fermentation setup is also shown for three time points (yellow circles). Values are shown as the mean ± standard deviation (*n* = 3). (B) Linear correlation between the ethanol concentration calculated from CO_2_ loss, and the ethanol concentrations determined by HPLC analysis of different fermentation experiments (*n* = 77).

To develop a simple and small-volume method for determination of ethanol production, we designed and evaluated the use of weight-loss measurement of small fermentation vials. In a fermentation of carbohydrates to ethanol, the production of ethanol correlates with the production of CO_2_, ideally in a 1:1 molar ratio (see the “Materials and methods” section). Hence, the weight loss of the fermentation system due to loss of gaseous CO_2_ is a measure of ethanol production. Figure 1 shows the vial design in which CO_2_ is released through a thin needle while preventing O_2_ from entering the vial. We observed that the ethanol production in glucose fermentation trials correlated with the vial’s weight loss (Fig. 2A). Based on multiple fermentation experiments (*n* = 77), we found a clear correlation between ethanol production determined by weight loss due to CO_2_ and ethanol production determined by HPLC analysis (Fig. 2B): Linear regression resulted in a slope of 1.132 (95% confidence interval: 1.101 to 1.163), a *y*-intercept of −0.222 (95% confidence interval: −0.765 to 0.321), and a coefficient of determination of *R*^2^ = 0.987. The results clearly showed a linear correlation between gravimetrically determined ethanol production and ethanol production determined by HPLC, which confirmed that the gravimetric method reliably estimated ethanol production.

### Preparation of cyanobacterial biomass

We hypothesized that the nitrogen content of the cyanobacterial biomass is important for its value as a nutrient supplement for yeast fermentation because a high nitrogen content in cyanobacteria typically correlates with a high content of protein and other nutrients (Forchhammer and Schwarz 2018). Under nitrogen limitation, cyanobacteria exhibit chlorosis during which the cells degrade proteins, pigments, nucleic acids, and other cellular components and exhibit a significantly increased C/N ratio. Therefore, to produce cyanobacterial biomass with different nitrogen contents and nutrient compositions, *Synechococcus* was cultivated under either N-replete or N-limited conditions (Fig. S1) to produce N-high and N-low biomass, respectively. The cultures were harvested after around 65 h, at which time the N-limited cultures exhibited chlorosis. As expected, the N-low biomass had significantly lower chlorophyll and phycobiliprotein contents and higher carbohydrate content than the N-high biomass (Table 1). The biomass was treated by either acid or enzymatic hydrolysis, as described in the “Materials and methods” section, prior to usage in the fermentation trials (Table 2). These treatments were selected because of their established use in biomass and bacterial cell degradation. Acid treatment represents a harsh approach, while lysozyme treatment represents a milder approach that presumably preserves biological molecules to a larger degree than acid treatment.

**Table 1.** Composition of cyanobacterial biomass.

Component	High-N biomass (*n* = 2)	Low-N biomass (*n* = 4)
Carbohydrate (% of dry cell mass)	16 ± 11	44 ± 23
Chlorophyll (arbitrary units^a^)	0.35 ± 0.01	0.18 ± 0.02
Phycobiliprotein (arbitrary units^a^)	0.28 ± 0.00	0.05 ± 0.03

a As defined by Möllers et al. (2014).

**Table 2.** Overview of cyanobacterial biomass supplements to *S. cerevisiae* fermentations.

Supplement	Cyanobacterial biomass^a^	Post-harvest treatment^a^
#a	Low N	Lysozyme, 37°C plus 50°C
#b	Low N	Lysozyme, 37°C
#c	Low N	Acid hydrolysis, 100°C
#d	High N	Lysozyme, 37°C plus 50°C
#e	High N	Lysozyme, 37°C
#f	High N	Acid hydrolysis, 100°C

a See the “Materials and methods” section for details.

### Fermentation of glucose

**Figure 3.**
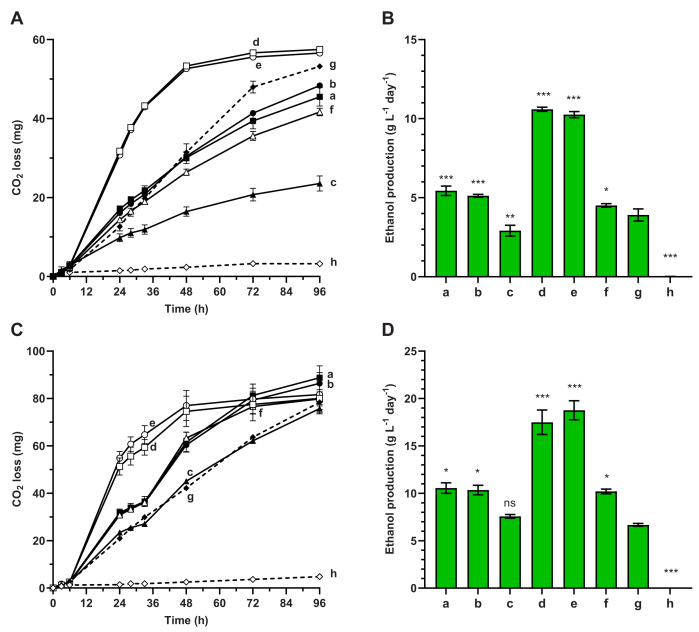
*Saccharomyces cerevisiae* fermentation of glucose (A and B) or wheat straw hydrolysate (C and D) supplemented with different *Synechococcus* hydrolysates (0.5% w/v) depicted as CO_2_-loss profiles (A and C) and the ethanol production rates in the first 24 h (B and D). Fermentations #g and #h refer to fermentations with and without yeast inoculum, respectively. For a description of other supplements, see Table 2. Fermentations in CBS medium with and without yeast inoculum were performed as positive control for yeast viability and as negative control to assess water evaporation, respectively (not shown). The highest fermentation rates were obtained with a supplement of high-nitrogen, lysozyme-treated *Synechococcus* biomass (#d and #e). The data are shown as mean ± standard deviation (*n* = 2–3). In panels B and D, differences are tested by ANOVA (*P*-values are adjusted for multiple comparisons to #g) and adjusted *P*-values are visualized as follows: *** <.0001; ** <.001; * <.05; ns = not significant).

To test the *S. cerevisiae* fermentation system with an optimal fermentation substrate, medium containing glucose (40 g l^−1^) was supplemented with differently pretreated cyanobacterial biomass (0.5% w/v) with either high or low nitrogen content (Tables 1 and 2). Figure 3A shows 96-h fermentation time-course experiments, and Fig. 3B shows the initial fermentation rates determined within the first 24 h. The highest increase in the fermentation rate, compared with the control with no addition of cyanobacterial biomass (#g), was obtained with lysozyme-treated, high-N cyanobacterial biomass (#d and #e), which resulted in about a 3-fold increase. Low-N (#a and #b) or acid-treated cyanobacterial biomass (#c and #f) had much less effect on the fermentation rate. Acid-treated, low-N cyanobacterial biomass (#c) even caused a slight decrease in fermentation rate, suggesting that yeast-inhibitory compounds were produced because of the acid treatment. These results confirm that cyanobacterial biomass can be used as supplements for yeast fermentations to enhance ethanol production from glucose and that enzyme treatment is superior to acid treatment for nutrient mobilization.

### Fermentation of wheat straw

Wheat straw represents a typical substrate for bioethanol fermentation that shows improved fermentation upon addition of synthetic or complex nutrients such as yeast extract (Jørgensen 2009). To test whether cyanobacterial biomass can substitute for these nutrients, wheat straw hydrolysates were supplemented with differently pretreated cyanobacterial biomass (0.5% w/v) with either high or low nitrogen content (Tables 1 and 2) and subjected to fermentation by *S. cerevisiae*. Figure 3C provides 96-h fermentation time-course experiments. The initial fermentation rate was enhanced for all fermentations with supplements when compared to the control fermentation #g with no supplements, except fermentation #c (low-N, acid-treated), which showed an almost identical fermentation profile to the control. Fermentations with supplements #d and #e (high-N, lysozyme-treated) reached their maximum CO_2_ loss of 75 and 77 mg, respectively, after 48 h with only little to no increase after, whereas it took 72 h for fermentations with supplements #a and #b (low-N, lysozyme-treated) and #f (high-N, acid-treated) to reach approximately the same values. Supplement #c and control fermentation #g reached their maximum CO_2_ loss after 96 h where all fermentations ceased at approximately the same amount of released CO_2_ (ca. 80 mg). In agreement with the CO_2_-loss measurements, the initial ethanol production rates were highest for fermentations with supplements #d (17.5 ± 1.3 g ethanol l^−1^ day^−1^, *n* = 2) and #e (18.7 ± 1.0 g ethanol l^−1^ day^−1^, *n* = 2), which corresponds to an increase of ∼3-fold compared to the control fermentation without nutrients (#g, 6.7 ± 0.2 g ethanol l^−1^ day^−1^, *n* = 3) (Fig. 3D). Interestingly, supplements #a, #b and #f showed almost identical ethanol production rates with 10.6 ± 0.5, 10.4 ± 0.5, and 10.3 ± 0.2 g ethanol l^−1^ day^−1^ (all *n* = 2), whereas supplement #c (7.7 ± 0.2 g ethanol l^−1^ day^−1^, *n* = 2) showed no significant increase in ethanol production compared to the control.

These results show that cyanobacterial biomass can indeed be used as supplements by yeast to enhance the fermentation of wheat straw. Furthermore, it is evident that high-nitrogen biomass is better suited to supplement wheat straw fermentations with an observed 30%–80% higher ethanol production rate compared to the average of all low-nitrogen content supplements. The lower ethanol production rate with N-low compared to N-high biomass is likely due to the fact that, under N-limited cultivation of cyanobacteria, an extensive physiological rearrangement and degradation of cellular components (phycobilisomes, ribosomes, thylakoids, etc.) takes place in order to adapt to and survive the physiological challenge of nitrogen limitation (Forchhammer and Schwarz 2018). In addition, the lysozyme-pretreated supplements performed consistently better than the acid-hydrolyzed supplements, but there was no significant difference between the two different lysozyme treatments. This observation emphasizes the use of enzymes rather than acid for degrading cyanobacterial cells and mobilization of the nutrients. It is interesting to notice that the high-nitrogen, acid-treated supplement #f showed a nearly identical rate compared to the low-nitrogen, enzyme-treated supplements #a and #b, suggesting that acid treatment causes a deterioration of valuable nitrogen sources/nutrients and reduces their ability to be used by yeast.

### Fermentation of spruce

**Figure 4.**
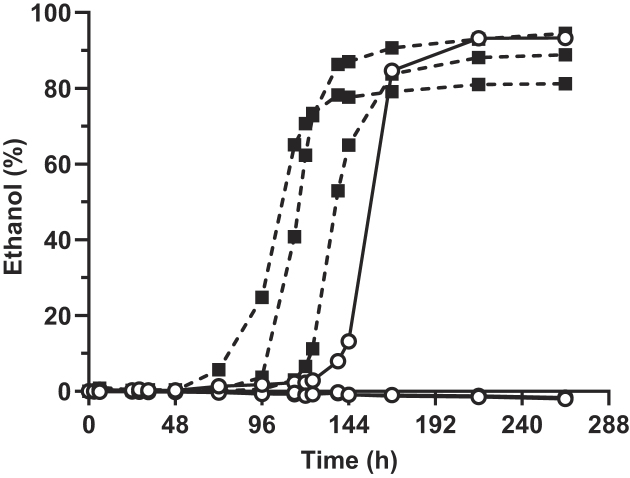
Time-course ethanol production during fermentations of spruce hydrolysates with high-nitrogen, enzymatic hydrolyzed *Synechococcus* biomass (1.5% w/v) (black squares with dashed lines) or a vitamin supplement identical to that used for CBS medium (white circles with full lines) (see the “Materials and methods” section). For each fermentation condition, three biological replicates were performed, and the ethanol production is shown for each replicate. 100% ethanol corresponds to a perfect stoichiometric conversion of glucose to ethanol. Ethanol production was not observed in the absence of nutrient supplements (not shown). Fermentations in CBS medium with and without yeast inoculum were performed as positive control for yeast viability and as negative control to assess water evaporation, respectively (not shown).

During the wheat straw fermentation, we identified the most beneficial cyanobacterial supplement as the high-N, lysozyme-treated biomass. This was then used as a supplement for fermentation of spruce hydrolysates at a loading of 1.5% (w/v), replacing the vitamin supplement usually used in CBS medium (see the “Materials and methods” section). As expected, the spruce fermentation without nutrients showed no ethanol production within the 264-h test period (data not shown). Ethanol production in the fermentations with cyanobacterial supplement was detected after 72 h in one batch and reached 50% of the theoretical maximum conversion in all three batches after ∼100–140 h (Fig. 4). Among the controls with CBS nutrients, one batch reached 50% ethanol of the theoretical maximum conversion after ∼160 h, whereas the remaining two batches did not produce any ethanol during the 264-h test period. Despite the described earlier onset of fermentation of the cyanobacterial supplemented fermentation, both types of fermentations reached the similar values after 168 h of incubation with ∼84% ethanol of the theoretical maximum conversion.

These results show that cyanobacterial supplements can be of substantial benefit in terms of fermentation onset and are advantageous during the first 72 h after onset (until 144 h). Even after detoxification, not all toxins are removed, which explains the generally long lag phase for spruce fermentations. It is possible that some of the residual toxins were scavenged by compounds in the cyanobacterial supplements, such as reactive oxygen species scavengers and other antioxidants (e.g. glutathione, carotenoids, and polyphenols; Guerreiro et al. 2020). The rapid slowdown after 144 h indicates that the available protein and nutrients were depleted by that time, suggesting that 1.5% (w/v) supplement may not be enough to observe the entire potential of cyanobacterial supplements. In addition, the initial lag phase of ∼72 h prior to ethanol evolution indicates a prolonged adaptation of the yeast cells, which is likely due to inhibitors present in hydrolyzed spruce material (Table S1).

## Discussion

The utilization of renewable resources such as plant biomass and photosynthetically produced microbial biomass is a promising strategy for developing sustainable processes to reduce greenhouse gas emissions. In this study, we demonstrated that pretreated whole-cell cyanobacterial biomass significantly enhances ethanol fermentation of lignocellulosic substrates by *S. cerevisiae*. Specifically, ethanol production rates from wheat straw increased ∼3-fold with high-nitrogen cyanobacterial hydrolysate produced by lysozyme treatment, while acid-hydrolyzed biomass yielded only a 0.5-fold increase. Low-nitrogen cyanobacterial biomass had minimal effect on fermentation, regardless of pretreatment. Fermentation of spruce hydrolysate also benefited from cyanobacterial supplementation, particularly in shortening the lag phase of fermentation.

We used a cyanobacterium to prepare the nutrient supplement because the cell wall of these organisms is easily hydrolyzed by lysozyme, thereby allowing a relatively mild treatment that preserves and mobilizes many cellular components (Möllers et al. 2014). In contrast, microalgae, being eukaryotes, possess diverse and often recalcitrant cell wall structures that complicate degradation and mobilization of cellular components (Shivakumar et al. 2024). We also tested acid treatment, which is a commonly used method for mobilization of biomass for biofuel fermentations, although this method tends to degrade many biomolecules valuable for growth (Markou et al. 2013). The cyanobacterial strain, *Synechococcus* sp. PCC 7002, was used because it is a fast-growing model cyanobacterium for which a multitude of research tools and literature is available (Cassier-Chauvat et al. 2021). Wheat straw and spruce were used as the lignocellulosic feedstocks, as they represent readily available agricultural and woody biomass distributed worldwide (Xiros et al. 2017, Taghizadeh-Alisaraei et al. 2022). Finally, we assessed the reliability of a simple milliliter-scale fermentation vial system based on gravimetric determination of ethanol production. The simplicity and small scale of this system enabled easy replication and testing of several experimental conditions. Furthermore, this system can be used in research and teaching settings without requiring costly analytical equipment such as HPLC or GC.

The feasibility of using cyanobacteria as a nutrient source at an industrial scale depends on both economic and practical considerations. In our experiments, cyanobacterial hydrolysate was effective when added to lignocellulosic biomass at a ∼1:70 mass ratio. This is comparable to previous findings using yeast extract, which was optimal at ∼1:285 (Jørgensen 2009). Based on this, a ratio of ∼1:100 could be sufficient for nutrient supplementation in industrial fermentations. Individual commercially operating biorefineries typically process 100 000 tons or more of lignocellulosic biomass per year by fermentation (Annevelink et al. 2022), which would correspond to a need for 1000 tons or more of cyanobacterial biomass per year. Although large-scale production of algae and cyanobacteria is still being developed, it is commercially viable at present. For example, the cyanobacterium *Arthrospira* is produced photosynthetically in open ponds at a scale of ∼12 000 tons annually, mostly in China (Qin et al. 2023). The cost of producing microalgal or cyanobacterial biomass in tubular photobioreactors with artificial light can exceed 50 € kg^−1^ but the costs in open raceway systems with natural illumination can be done at roughly 5 € kg^−1^ (Fernandez et al. 2019). If wastewater is used for cultivation in an open raceway, the costs can get below 2 € kg^−1^. Therefore, integrating cyanobacterial production within a biorefinery setting where multiple outputs and resource recycling are possible may help reduce costs and improve feasibility. However, certain regulatory constraints must also be considered, such as the use of genetically modified cyanobacteria or cultivation in contaminated wastewater, which could be problematic if pathogens, heavy metals, or other pollutants are present.

Future research should address how best to mobilize the fermentation-enhancing nutrients from cyanobacterial cells in a cost-effective manner. Our results indicate that lysozyme treatment is more effective than acid hydrolysis, likely because it preserves bioactive compounds. Other strategies that could be explored to improve the scalability of cell lysis include native autolysis (Demirkaya et al. 2023; Zorz et al. 2023), engineered lysis (Miyake et al. 2014), and chemical lysis (Mehta et al. 2015). Future work should also identify the specific compounds in cyanobacterial hydrolysate that enhance *Saccharomyces* fermentation. These may include vitamins, amino acids, and stress-protective compounds. Profiling yeast transcriptomes or testing defined compound mixtures could help determine these active components. In addition, optimizing yeast strains for improved uptake and utilization of nutrients from cyanobacterial hydrolysates could further enhance fermentation efficiency.

In conclusion, this study demonstrates that cyanobacterial biomass can serve as a sustainable and effective alternative to conventional yeast extract supplements for ethanol production from lignocellulosic substrates. Depending on cultivation conditions and pretreatment methods, cyanobacterial biomass can be used as a source of fermentable carbohydrates, as a nutrient supplement, or as a provider of value-added bioproducts, fitting well into integrated biorefinery frameworks.

## Supplementary Material

qvaf007_Supplemental_File

## Data Availability

The data underlying this article are available in the article and in its online supplementary material.
