# Supplementary material for: Whole-cell hydrolysate of cyanobacteria enhances biofuel fermentation of plant biomass
Source: Sustain Microbiol. 2025 May 1;2(2):qvaf007. doi: 10.1093/sumbio/qvaf007 (PMC13375342; doi:10.1093/sumbio/qvaf007)
Supplement: Supplementary file 1 [file qvaf007_Supplemental_File.pdf]

Supplementary Figure S1

Cultivation of *Synechococcus* under N-replete conditions (2.0 g $NaNO_3$ L^{-1}, open symbols) and under N-limited conditions (0.24 g $NaNO_3$ L^{-1}, closed symbols). **(A)** Total carbohydrate per cell dry weight (% w/w). **(B)** $NaNO_3$ consumption. **(C)** Phycobilisomes (PBS, squares) and chlorophyll *a* (Chl *a*, circles) content (arbitrary units).

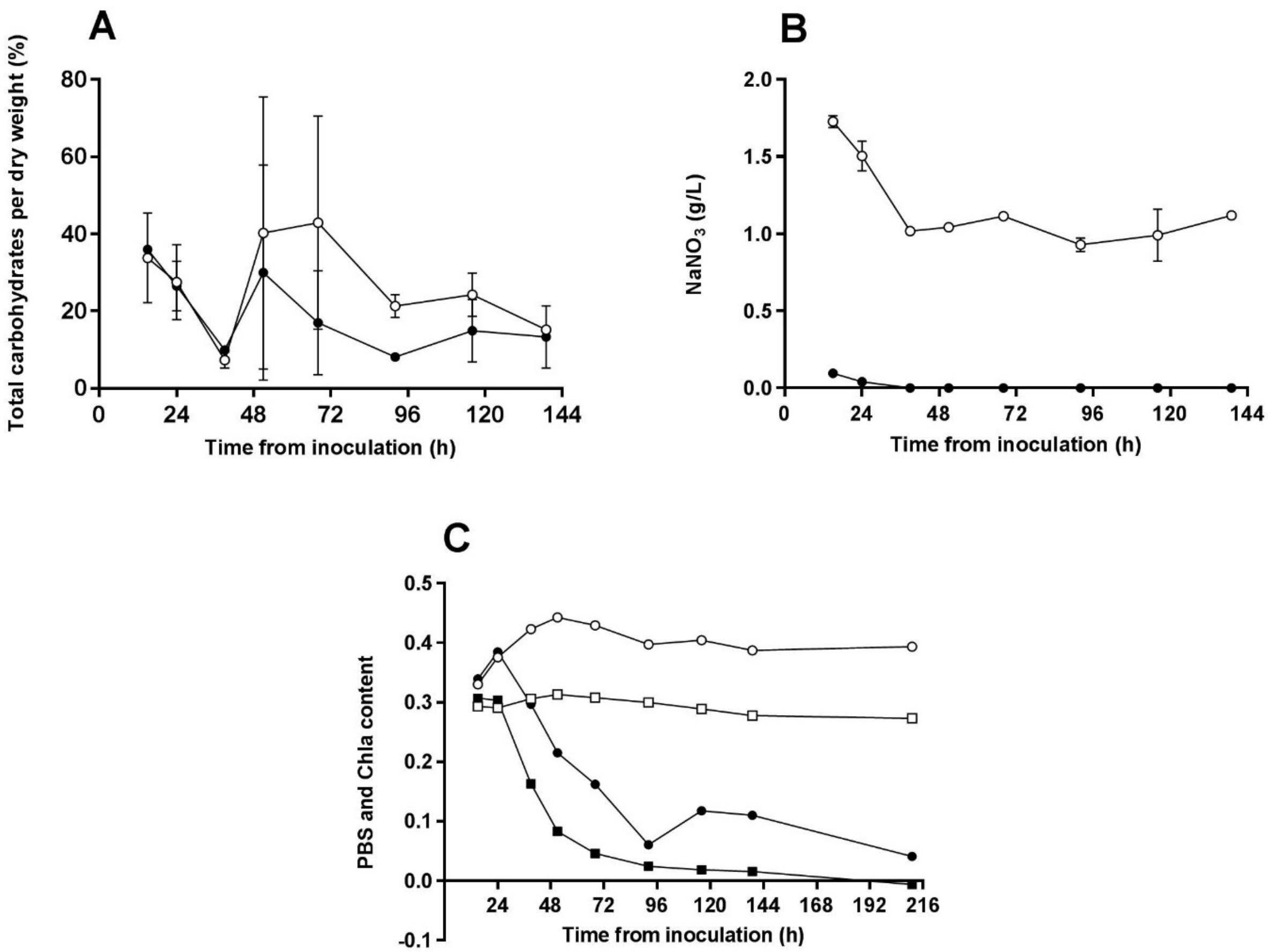

Supplementary Table S1

Composition of spruce hydrolysates before and after PEI treatment.

Sample	Furfural (g L^{-1})	HMF (g L^{-1})	Acetate (g L^{-1})	Glucose (g L^{-1})
**Hydrolysates ÷ PEI**	2.21	4.63	8.11	99.13
**Hydrolysates + PEI**	0.97	2.21	5.62	70.79
